# Circulating Nitric Oxide and Metabolic Syndrome in Arab Children and Adolescents: A Case–Control Study

**DOI:** 10.3390/children10020210

**Published:** 2023-01-25

**Authors:** Osama E. Amer, Shaun Sabico, Malak N. K. Khattak, Nasser M. Al-Daghri

**Affiliations:** Biochemistry Department, College of Science, King Saud University, Riyadh 11451, Saudi Arabia

**Keywords:** cardiovascular diseases, diabetes mellitus, Saudi, sexual dimorphism, dyslipidemia

## Abstract

Background: Metabolic syndrome (MetS) represents a cluster of known cardiometabolic risk factors, which elevates the risk of type 2 diabetes mellitus (T2DM), atherosclerotic cardiovascular disease (CVD) and chronic kidney disease (CKD) in adults and, only recently, even in children and adolescents. Circulating nitric oxide (NOx) has been observed to influence MetS risk factors in adults, but this has been scarcely investigated in children. The aim of the present study was to determine whether circulating NOx levels correlate with known components of MetS in Arab children and adolescents. Methods: Anthropometrics, serum NOx, lipid profile and fasting glucose levels were measured in 740 Saudi Arabs aged 10–17 years (68.8% girls). The presence of MetS was screened using the criteria of de Ferranti et al. Results: Overall, serum NOx levels were significantly higher in MetS participants compared to non-MetS (25.7 µmol/L (10.1–46.7) versus 11.9 µmol/L (5.5–22.9), *p* < 0.001) even after adjustments for age, BMI and sex. With the exception of elevated blood pressure, higher circulating NOx significantly increased the odds for MetS and its components. Lastly, receiver operating characteristics (ROC) showed that NOx, as a diagnostic marker for MetS, had good sensitivity and was higher in boys than girls (all MetS participants: area under the curve (AUC) = 0.68, *p* < 0.001), (girls with MetS: AUC = 0.62, *p* = 0.002), (boys with MetS: AUC = 0.83, *p* < 0.001)). Conclusions: MetS and most of its components were significantly associated with circulating NOx levels in Arab adolescents and may be a promising diagnostic biomarker for MetS.

## 1. Introduction

Metabolic syndrome (MetS) is a global epidemic with the hallmark clinical presence of a low-grade chronic inflammatory state secondary to the clustering of multiple metabolic risk factors for type 2 diabetes mellitus (T2DM), atherosclerotic cardiovascular disease (CVD) and even chronic kidney disease (CKD) [1,2]. It has been observed that MetS starts early in life, long before the occurrence of clinical disease, which, if left untreated, persists into adulthood [3,4]. Collectively, the risk factors for MetS include obesity, glucose intolerance or T2DM, dyslipidemia (hypertriglyceridemia and/or low high-density lipoprotein cholesterol, HDL-C) and elevated blood pressure or hypertension [5].

The growing prevalence of childhood obesity has been established as the primary cause of worldwide rise in the prevalence of MetS in this population [6,7]. In Saudi Arabia, obesity is highly prevalent in both children (20.6%) and adults (39.8%) [8,9]. Persistence of MetS in childhood elevates susceptibility to premature atherosclerosis and CVD in early adulthood [3,5]. In a recent longitudinal study [10], childhood MetS was a significant predictor of CVD even 25 years later in adulthood. Moreover, the risk factors for having CVD were shown to be not only hereditary but persisting throughout adulthood [11,12,13].

Nitric oxide (NOx) is a pleiotropic signaling molecule produced by the endothelium and is present in almost every cell type [14]. NOx and its metabolites, nitrate and nitrite, have diverse roles in human metabolism, such as inflammation, oxidative stress, vasodilation, cardiac function, reproduction, gene transcription and translation and post-translational modifications [15,16,17,18]. Although limited in causality, there is strong evidence of the associations between circulating NOx and MetS in adults based on several observational studies [19,20,21]. Consequently, the significant associations of serum NOx levels with childhood obesity and lipids have been reported, including those with type 1 diabetes mellitus T1DM [22,23,24]. However, studies on the associations of NOx and cardiometabolic parameters in children are inconsistent, and this can be partially attributed to the different ethnicities of the populations used in investigations, as well as differences in the assays used for the assessment of NOx. Nevertheless, a pathophysiological mechanism of NOx in diabetes could be its close association with insulin resistance and endothelial dysfunction, which in turn links metabolic disorders demonstrated by MetS with cardiovascular homeostasis [25]. Moreover, previous studies have demonstrated that the production of NOx at high levels leads to pathological alterations in several metabolic pathways [26,27].

Given the heightened concern over the growing emergence of MetS and its components in pediatric populations of different ethnicities, data on several biomarkers and environmental factors associated with childhood MetS may provide additional insights into the mechanisms of this chronic disorder [28]. Arguably, there is limited investigation on the associations of circulating NOx with pediatric MetS. Therefore, the present study aims to investigate and determine the associations of circulating NOx levels with MetS and its corresponding elements in a cohort of Arab children and adolescents in a school setting.

## 2. Materials and Methods

### 2.1. Participants

In the present multi-center cross-sectional study, a total of 740 apparently healthy school-attending Saudi children and adolescents aged 10–17 years (68.8% girls) were recruited from the school project master database of the Chair for Biomarkers of Chronic Diseases (CBCD) at King Saud University (KSU), Riyadh City, Saudi Arabia. Written informed consent was obtained from parents and assent from students. All experiments and data collection methods were seen and approved by the Institutional Review Board (IRB) of the College of Science, KSU, Riyadh, Kingdom of Saudi Arabia (IRB Ref No 15/0502/IRB dated 21 December 2015), and were conducted in one central laboratory (CBCD, KSU). All procedures were performed in full accordance with the Helsinki Declaration of 1964 ethical standards and its recent amendments.

### 2.2. Anthropometrics and Biochemical Analyses

All students were instructed to be in a 10 h overnight fasting state. On an assigned date approved by the school principal, trained nurses collected the fasting blood samples and anthropometric measurements at the school site. Coordination with the school authorities was necessary, so that mass data collection could be implemented in a shorter period of time without inflicting much inconvenience on both the class schedules and students. Fasting blood samples were centrifuged immediately on site; the serum was separated in plain tubes, kept in ice-filled containers, and immediately transported to the central laboratory, CBCD, KSU, Riyadh, Saudi Arabia, where all blood and serum samples were stored at −80 °C. Anthropometrics were measured and included height (cm), weight (kg), hip (cm) and waist circumference (WC) (cm). Body mass index BMI (kg/m^2^) was calculated. Blood pressure (mmHg) (systolic, SBP, and diastolic, DBP) was noted as the average of two readings with a 15 min interval, using a pediatric cuff if applicable. A standard routine laboratory analysis (Konelab, Espoo, Finland) was used to measure fasting blood glucose (mmol/L), total cholesterol (TC) (mmol/L), triglycerides (TAG) (mmol/L) and HDL-C (mmol/L). This biochemical analyzer was calibrated regularly prior to the analysis of all serum samples using quality control samples purchased from the manufacturer. The limit of detection (LOD) for the assay was 0.02 mmol/L (for glucose), 0.1 mmol/L (for TC), 0.04 mmol/L (for HDL-C) and 0.02 mmol/L (for TAG). The inter-assay CVs were ≤5% (for glucose), ≤3.5% (for TC), ≤4% (for HDL-C) and ≤4% (for TAG) for these tests, respectively. The Friedwald equation was used for obtaining LDL cholesterol (LDL-C) (mmol/L) values. Circulating serum NOx (µmol/L) was measured using the Griess reaction [29]. The obesity status was classified according to the participant’s BMI percentile, age and sex, as done previously [8,30].

### 2.3. Definition of Pediatric MetS

The De Ferranti et al. 2004 criteria were used to define MetS in the present study population [31] due to their common use in epidemiological studies, the ability to capture a higher yield of high-risk children, the lack of prerequisite risk factors, such as central obesity or elevated glucose levels as the primary component, and their convenience for mass screening in select community populations as compared to other definitions [32].

Participants who presented with three risk factors or more were considered to have pediatric MetS:

1. Hypertriglyceridemia, defined as triglyceride levels ≥ 1.1 mmol/L.
2. Low HDL-C < 1.3 mmol/L (for boys 15–19 years, HDL-C < 1.17 mmol/L).
3. Elevated fasting blood glucose ≥ 6.1 mmol/L.
4. Central obesity, defined as >75th percentile of waist circumference based on age and gender.
5. Elevated blood pressure, defined as systolic or diastolic blood pressure (mmHg), which is >90th percentile for age, sex and height.

### 2.4. Data Analysis

Data were analyzed using SPSS (version 22.0, IBM, Chicago, IL, USA). Post hoc power analysis (1-β) = 0.805 was performed using G*Power (3.1) with effect size d between group = 0.25, α error of probability = 0.05 and sample size (control = 620 and MetS = 120), respectively. Continuous parameters were shown as mean ± standard deviation (SD) for normally distributed variables, while variables that were not normally distributed were shown as median (first and third quartiles). Categorical parameters were presented as frequencies and percentages (%). All continuous variables underwent assessment for normality using the Kolmogorov–Smirnov test. Non-normal variables were log-transformed prior to the parametric analysis. The independent *t*-test and Mann–Whitney U-test were used to compare the mean and median differences in normal and non-normal variables, respectively, and univariate analysis was performed for covariate adjustments, which included age, BMI and sex. Multinomial logistic regression analysis was performed for MetS and its components, and receiver operating characteristic (ROC) curve was used to determine the sensitivity and specificity of serum NOx in diagnosing MetS. A *p*-value < 0.05 was considered statistically significant.

## 3. Results

Table 1 shows the differences in cardiometabolic characteristics of non-MetS versus MetS participants in boys and girls. The overall prevalence of MetS was 16.2% (120 out of 720). The prevalence of MetS in girls was 15.6% (77 out of 495), while the prevalence of MetS in boys was slightly higher at 17.6% (43 out of 245). In all participants and as expected, the MetS group had a significantly higher BMI Z-score, WC, SBP and DBP than the non-MetS group, even after adjusting for age, BMI and sex (*p*-values < 0.001). Among the biochemical parameters, only TC was not significantly different in both groups (*p* = 0.53), while LDL-C was borderline significant in favor of the MetS group (*p* = 0.05). Fasting glucose and TAG were significantly higher in the MetS group (*p*-values 0.002 and <0.001, respectively), while HDL-C was significantly lower in the MetS group as compared to their non-MetS counterparts (*p* < 0.001). Lastly, the median levels of NOx were significantly higher in the MetS as compared to the non-MetS group, even after adjusting for covariates (25.7 µmol (10.1–46.2) versus 11.9 µmol (5.5–22.9); *p* < 0.001). When stratified according to sex, in girls, the majority of the variables mentioned remained significant even after adjusting for age and BMI, with the exception of WC (*p* = 0.051), TC (*p* = 0.85) and LDL-C (*p* = 0.16). The median levels of NOx in girls with MetS were significantly higher compared to those without and remained persistent even after adjusting for covariates (12.0 µmol (6.7–30.6) versus 8.7 µmol (4.8–18.6); *p* = 0.002). Lastly, in boys, only TC (*p* = 0.84), HDL-C (*p* = 0.34) and LDL-C (*p* = 0.20) were not significantly different in MetS versus non-MetS groups. Similar to girls, the median level of NOx was significantly higher in the MetS than the non-MetS group after adjustment (42.3 µmol (31.4–62.9) versus 18.9 µmol (11.2–31.7); *p* < 0.001). It is worth noting that the NOx levels in boys without MetS were still significantly higher compared to girls with MetS (18.9 µmol (11.2–31.7) versus 12.0 µmol (6.7–30.6); *p* < 0.05) and that the NOx levels in boys were significantly higher than in girls, independent of MetS status. The rest of the comparisons are presented in Table 1.

Table 2 shows the odds ratios for MetS and its components in NOx tertiles. The results showed that having higher tertiles of serum levels of NOx is a significant risk factor for MetS as compared to those in the lower tertile, with an odds ratio (OR) of 3.63 (95% confidence interval, CI, 2.12–6.19), *p* > 0.001. The odds increased and remained significant after adjusting for age and sex (OR 3.84 (95% CI 2.12–6.92), *p* < 0.001). In terms of the influence of varying NOx serum levels on individual MetS components, those with serum levels of NOx in the higher tertile faced a risk factor for high WC (OR 1.76 (95% CI, 1.07–2.91), *p* = 0.026), high blood glucose (OR 2.48 (95% CI, 1.18–5.21), *p* = 0.016) and high TAG (OR 2.33 (95% CI, 1.54–3.53), *p* < 0.001) compared with those in the lower tertile. Serum levels of NOx in the second or third tertiles were risk factors for low HDL-C levels (OR for second tertile: 2.62 (95% CI, 1.74–3.94), *p* < 0.001 and third tertile: OR 6.2 (95% CI, 3.9–9.9), *p* < 0.001) compared to those in the lower tertile, even after adjusting for age and sex. The rest of the ORs are found in Table 2.

**Figure 1:**
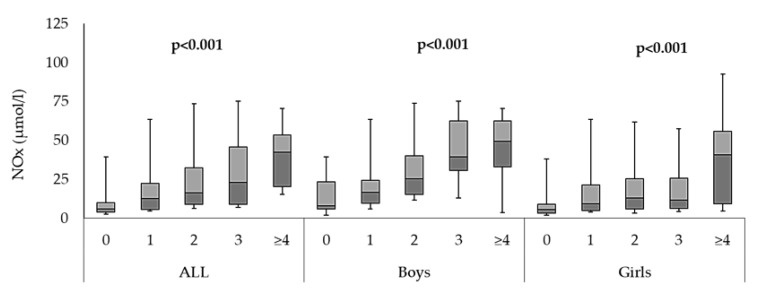
Comparison of serum NOx (µmol/L) levels and of clustering of MetS individual components in all participants, as well as in boys and girls.

Participants were further stratified based on the clustering of MetS components and sex. These included the five MetS components: central obesity, hyperglycemia, hypertension, low HDL-C and hypertriglyceridemia. The results showed that NOx levels were significantly higher in participants with increasing number of MetS components compared to healthy participants with no MetS risk factor in both sexes (*p* < 0.001) (Figure 1).

**Figure 2:**
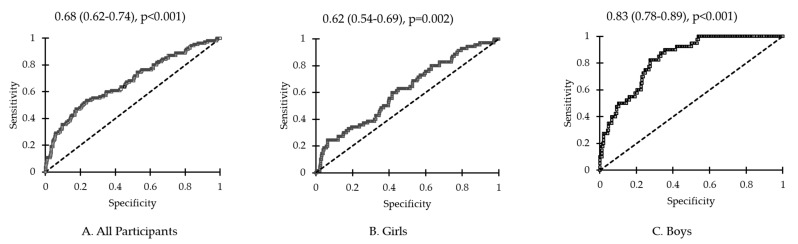
Receiver operating curve for NOx in (**A**) all participants, (**B**) girls and (**C**) boys.

Lastly, ROC analysis was performed to assess whether NOx could be a reliable biomarker for pediatric MetS. The area under the curve (AUC) showed that NOx had good sensitivity for MetS diagnosis, and it was higher in boys than in girls ((all MetS participants: AUC = 0.68, *p* < 0.001), (girls with MetS: AUC = 0.62, *p* = 0.002), (boys with MetS: AUC = 0.83, *p* < 0.001)) (Figure 2).

## 4. Discussion

The present study investigated serum levels of NOx in relation to MetS in a group of Saudi Arabian school children and adolescents and evaluated the relationship between NOx levels and cardiometabolic parameters according to pediatric MetS status. The present study is arguably the first to investigate pediatric MetS and its association with serum NOx levels among Arab children and adolescents. Remarkably, the results of the ROC analysis indicated an important role for NOx in MetS and that NOx could be a promising diagnostic biomarker for MetS, with higher sensitivity in boys than in girls. Interestingly, the prevalence of MetS in Saudi school children was high in both boys and girls, as previously documented, suggesting that pediatric MetS in the kingdom may soon be a public health threat, if it is not already so, unless proper interventions and public awareness campaigns are instigated on a national level [8].

In the present investigation and as expected, several cardiometabolic parameters assessed were significantly higher in the MetS group as compared to those without MetS even after adjusting for covariates, with the exception of HDL-C. These findings were true even after stratification for sex. More interestingly, serum NOx levels were observed to be significantly higher in participants with MetS than in those without MetS even after adjusting for age, sex and BMI, and also after stratification according to sex, with boys having higher NOx levels independent of MetS status. In addition, elevated NOx levels were a risk factor for MetS and its individual components in the present study cohort. Previous studies have demonstrated the associations between cardiometabolic factors and serum NOx; Higashino et al. documented a direct association between plasma NOx levels and blood pressure among African Americans with comorbid diseases and proposed that assessment of serum NOx could help in monitoring hypertension state and severity [33]. In another study conducted in Italy, circulating NOx production was increased in diabetes patients with MetS, as defined using the International Diabetes Federation (IDF) criteria, as well as those with high TAG/HDL-C index [21], which may be secondary to the over-stimulation of inducible NOS (iNOS) mRNA in the pancreatic islets and elevated glycosylation end products [34]. With regard to lipids, it was previously demonstrated that NOx production, at least in the context of basal endothelium synthesis, was inversely associated with elevated cholesterol states or dyslipidemia in humans [35]. Within the context of adiposity, NOx synthases, endothelial NOS (eNOS) and iNOS, have been shown to present in adipose tissue and to be influenced by lipolysis of subcutaneous tissue independent of obesity status, suggesting that NO can regulate adipose tissue through inhibition of lipolysis [36]. Mechanistic investigations found that the iNOS protein is present in human subcutaneous adipose tissue and demonstrated that iNOS inhibition had led to increased lipolysis in this tissue [37,38].

The present study results identified NOx as a risk factor for MetS and its components (Table 2). These results show a relationship between serum NOx and MetS in our pediatric cohort, which was reported previously in adults of different backgrounds, including Amish women [20], Italian adults [21] and obese children of south Asian origin [39]. Additionally, serum NOx was directly proportional to the number of MetS components (Figure 1), which is in line with a previous study in adults showing increased serum NOx levels with increasing individual components of MetS in both the main analysis and the sex-specific sub-analysis [20]. NOx is biosynthesized in several cell types other than the vascular endothelium; therefore, it could be speculated that increased serum NOx in MetS, as seen in our results, could be due to eNOS inhibition and iNOS overexpression, as recently observed [21]. Evidence supporting this relationship has been reported previously. Maejima et al. [40], in their case–control study involving hospitalized patients with and without diabetes, found significantly higher NOx serum levels in hypertensive diabetic patients compared to normotensive patients with diabetes, suggesting that, again, impaired NOx activities are affected by the weakness of the basal endothelium vasodilation, leading to elevated oxidative stress. Since the elevation of circulating NOx is significantly influenced by the strength of endothelium, and the weakness of the endothelium is exacerbated by increasing number of MetS components, it makes sense to assume that the relationship between NOx and the clustering of MetS risk factors is direct and parallel.

NOx is generated at different rates by different NOx synthases isoforms, and contrary to popular belief that free radicals are unstable, NOx is actually chemically stable and is only substantially affected by the presence of other free radicals, such as superoxides [14,41]. The inducible NOS or iNOS isoform, expressed in response to inflammatory stimuli in several types of cells, has the largest capacity to produce NOx [42,43]. Obesity is associated with notable changes in the abundance of the iNOS enzyme isoform, which abundantly increases in adipose tissue [44] and skeletal muscle [45] in obese states. Tumor necrosis factor alpha (TNF-α) is an inflammatory adipocytokine, which was consistently demonstrated to be significantly increased in individuals with MetS compared to normal ones, supporting the fact that inflammation has an important role in MetS pathogenesis [46,47]. Moreover, it has been previously observed that TNFα is associated with NOx synthesis activity through a variety of pathways, most commonly through the activation of Akt at Ser 473, a signaling pathway involved in several metabolic activities, including tumor growth and insulin resistance [48]. This could partially explain the elevated NOx levels in participants with MetS in the present study [49].

The present results showed sex differences in serum NOx levels between boys and girls, and this was true even among boys without MetS, with significantly higher NOx compared to girls with MetS. Alchujyan et al. [50] also observed differences in NOS activities in peripheral blood leukocyte cells in a group of children/adolescents and young adults with type 1 diabetes (T1D) and observed that several factors affect bioavailable NOx, which include not only glycemic status and duration of T1D but also age and sex. Additionally, Ratajczak et al. [51] found that the expression of iNOS was higher in female neutrophils than in males, and this was attributed to the physiological differences in circulating estrogen among the sexes. These sex differences could be a result of sex-specific variations or a difference in the ability of NOS gene expression depending on levels of androgens, estrogen in particular, or might also be ascribed to differences in the composition of the adipose tissues. Previously, it was demonstrated that more than 6500 protein-coding genes are differentially expressed in men and women, which results in sexually dimorphic properties in both sexes, which may also explain why the male sex on its own is a CVD risk factor [52].

The authors acknowledge some limitations, which need to be considered in the interpretation of the present results. First, the dietary intake of the participants was not recorded. It was observed that dietary-derived NOx was a contributor to NOx levels in apparently healthy participants, making it a potentially significant confounder, which can bias findings [53,54]. Dietary collection, however, is particularly difficult to implement in the present study, given the school setting and the absence of parental participation, which can largely contribute to the accuracy of the dietary recall of participants. Second, an evaluation of the inflammatory status was not performed, and this could have strengthened the diagnostic power and clinical value of NOx in terms of reaffirming why the level of increase is compounded with the parallel increase in MetS risk factors. Lastly, the present study implemented a cross-sectional design, and thus, the potential causality between NOx and other variables cannot be measured. Interventional studies with matched-controls are needed in which NOx and MetS components are monitored over time to ascertain whether varying NOx levels are the cause of the increase or decrease in metabolic parameters over time. Nevertheless, the present study provides novel information on circulating NOx levels among Saudi Arabian children and adolescents with or without MetS in Saudi Arabia. The findings are consistent with observations conducted in adult populations with MetS and other chronic diseases, such as CVD and T2DM.

## 5. Conclusions

Circulating serum NOx was significantly associated with MetS and its individual components in Saudi children and adolescents and was sexually dimorphic, suggesting that NOx may play a role in the metabolic status and the modulation of pediatric MetS similar to adults. Circulating NOX may be a promising marker for pediatric MetS, especially in boys. Follow-up studies that are interventional in nature, such as lifestyle modification programs and non-pharmacological clinical trials, are required to not only mitigate the increasing pediatric MetS among Arab adolescents but also to better understand the pathophysiological mechanisms of NOx and its potential applications in the prevention of premature CVD and DM in the pediatric population, especially in boys.

## Figures and Tables

**Table 1:** Clinical characteristics of participants.

**Parameter**	**All**	**All**		***p*-Value Adjusted for Age, BMI and Sex**	**Girls**		***p*-Value** **Adjusted for Age and BMI**	**Boys**		***p*-Value Adjusted for BMI**
		**Non-MetS**	**MetS**		**Non-MetS**	**MetS**		**Non-MetS**	**MetS**	
N (Boys/Girls)	740 (245/495)	620 (202/418)	120 (43/77)		418	77		202	43	
Age (years)	14.2 ± 1.6	14.1 ± 1.6	14.7 ± 1.7		14.1 ± 1.7	15.1 ± 1.8		14.1 ± 1.0	14.1 ± 1.2	0.80
BMI (kg/m^2^)	21.9 ± 4.8	21.3 ± 4.5	25.5 ± 4.7		20.8 ± 4.6	25.8 ± 4.7		22.1 ± 4.1	25.1 ± 4.7	
BMI Z-Score	0.00 ± 1.0	−0.137 ± 0.94	0.753 ± 0.97	<0.001	−0.232 ± 0.97	0.810 ± 0.97	<0.001	0.048 ± 0.87	0.669 ± 0.98	<0.001
WC (cm)	73.6 ± 11.7	71.5 ± 10.6	83.2 ± 11.9	<0.001	72.0 ± 11.2	86.8 ± 12.2	0.051	70.9 ± 9.6	78.2 ± 9.7	<0.001
SBP (mmHg)	119.4 ± 14.0	117.2 ± 12.8	130.3 ± 14.4	<0.001	118.9 ± 13.0	132.6 ± 14.7	<0.001	113.6 ± 11.8	126.3 ± 13.2	<0.001
DBP (mmHg)	70.1 ± 9.8	69.3 ± 9.3	74.5 ± 10.9	<0.001	68.3 ± 9.1	71.9 ± 10.1	0.004	71.2 ± 9.5	78.5 ± 11.1	<0.001
TC (mmol/L)	4.30 ± 1.03	4.29 ± 1.03	4.36 ± 1.01	0.53	4.43 ± 0.9	4.51 ± 0.9	0.85	4.01 ± 1.2	4.10 ± 1.1	0.84
Glucose (mmol/L)	5.13 ± 0.72	5.10 ± 0.7	5.40 ± 0.90	0.002	5.2 ± 0.6	5.5 ± 0.7	0.05	4.9 ± 0.7	5.2 ± 1.0	0.02
HDL-c (mmol/L)	1.18 ± 0.41	1.22 ± 0.42	0.97 ± 0.25	<0.001	1.32 ± 0.5	1.01 ± 0.3	<0.001	1.0 ± 0.2	0.92 ± 0.2	0.34
LDL-c (mmol/L)	2.63 ± 0.79	2.59 ± 0.8	2.79 ± 0.9	0.05	2.62 ± 0.8	2.82 ± 0.9	0.16	2.54 ± 0.8	2.74 ± 0.9	0.20
TAG (mmol/L) #	1.0 (0.8–1.4)	0.97 (0.75–1.3)	1.4 (1.1–1.8)	<0.001	0.97 (0.8–1.3)	1.4 (1.1–1.7)	<0.001	0.98 (0.7–1.2)	1.4 (1.2–1.8)	<0.001
NOx (µmol/L) #	12.8 (5.7–26.4)	11.9 (5.5–22.9)	25.7 (10.1–46.2)	<0.001	8.7 (4.8–18.6)	12.0 (6.7–30.6)	0.002	18.9 (11.2–31.7)	42.3 (31.4–62.9)	<0.001

Note: Data presented as N for frequencies; mean ± SD for normally distributed variables; # denotes non-normally distributed continuous variables, presented as median (25th–75th) percentiles; *p*-value significant at <0.05. BMI—body mass index; DBP—diastolic blood pressure; NOx—nitric oxide; SBP—systolic blood pressure; TAG—triglycerides; WC—waist circumference; TC—total cholesterol.

**Table 2:** Risk factors for MetS and its components according to NOx tertiles.

Parameters	Odds Ratio (95% CI)		Age- and Sex-Adjusted Odds Ratio (95% CI)	
MetS				
T1	1		1	
T2	1.41 (0.77–2.56)	0.26	1.18 (0.63–2.22)	0.61
T3	3.63 (2.12–6.19)	<0.001	3.84 (2.12–6.92)	<0.001
Waist (cm)				
T1	1		1	
T2	1.02 (0.63–1.65)	0.93	1.04 (0.62–1.74)	0.87
T3	1.61 (1.03–2.52)	0.038	1.76 (1.07–2.91)	0.026
Hypertension (mmHg)				
T1	1		1	
T2	1.14 (0.67–1.92)	0.63	0.89 (0.51–1.56)	0.68
T3	1.49 (0.90–2.46)	0.120	1.46 (0.84–2.53)	0.18
High Glucose (mmol/L)				
T1	1		1	
T2	1.38 (0.64–2.99)	0.41	1.17 (0.53–2.61)	0.70
T3	2.65 (1.31–5.32)	0.006	2.48 (1.18–5.21)	0.016
High TAG (mmol/L)				
T1	1		1	
T2	1.57 (1.07–2.32)	0.02	1.41 (0.93–2.12)	0.10
T3	2.33 (1.59–3.42)	<0.001	2.33 (1.54–3.53)	<0.001
Low HDL-C (mmol/L)				
T1	1		1	
T2	2.81 (1.92–4.10)	<0.001	2.62 (1.74–3.94)	<0.001
T3	7.77 (5.10–11.97)	<0.001	6.24 (3.93–9.87)	<0.001

Note: Data presented as odds ratio (95% confidence interval); T represents tertile; significance at *p* < 0.05.

## Data Availability

The data presented in this study are available on request from the corresponding author. The data are not publicly available due to privacy protection.
